# Clinic of the Jefferson Medical College

**Published:** 1844-05-04

**Authors:** Edward R. Squibb


### CLINICAL LECTURES AND REPORTS.

### PHILADELPHIA HOSPITAL.

CLINIC OF THE JEFFERSON MEDICAL COLLEGE.

*February* 21, 1844.

CONCLUDING LECTURE OF PROFESSOR DUNGLISON.

[Reported by Edward R. Squibb.]

TYPHOID FEVER.

The Professor first presented to the class a well-marked case of typhoid fever, by which to illustrate some observations which he desired to make upon this important disease before the close of the session.

The class, he said, were aware that, within the last few years, a division had been made in what was before regarded as typhus fever, into two distinct forms,—*typhus* and *typhoid*, based upon a distinction in the pathological lesions, and therefore in certain of the morbid phenomena. In this division, the celebrated M. Louis, of Paris, had particularly distinguished himself, by his care and skill in investigating and presenting what he considered to be the distinguishing traits of typhoid as compared with other fevers.

This distinction (which is not, however, admitted by British authorities in general) consists, as alleged by Louis, in the absence, in true typhus, of any intestinal lesion; whilst, in typhoid, there is generally —some say, universally—a well-marked affection of the follicles of the intestine, and most commonly in those patches of Peyer which are found in the ileum, near the colon. In typhus, petechiæ and vibices, or livid marks, such as would be produced by the stroke of a whip, are observed; which, being absent in typhoid, are usually counterbalanced by the occurrence, after the first week, of *taches rouges*, or rose spots, resembling flea bites. These disappear on pressure, but immediately recur on its removal.

Meteorism and enlargement of the spleen are also said to be usually present in the typhoid form. Both, however, are found to occur frequently in other affections.

These divisions, the lecturer thought, are not proven; and instead, therefore, of adopting the views of those who regard the follicular affection as the primary lesion, and, as such, productive of the phenomena observable in typhoid fever, he would rather regard it as an accidental difference in the expression of adynamic fever, or as a mere variety of the same disease as typhus. Thus, since the attention of the pathologists of Great Britain has been more directed to the diagnosis of the typhoid affection and typhus, it has been frequently discovered, that to the all ged pathognomonic symptoms of typhus have been added the intestinal lesion, meteorism, &c., of the typhoid affection. The Professor alluded, also, to cases occurring in this country, in which there was an evident mixture of the two. From all his own observations,and reflections based on the observations of others, he was not, therefore, prepared to admit typhoid and typhus to be two distinct diseases; he thought that both are forms of adynamic fever, presenting different phenomena under different circumstances. In this country, and in France, the intestinal affection is generally present; whilst in England, it would appear to be as commonly absent.

Such being his view, the principal indication in the treatment would be, to combat the pathological conditions as they presented themselves, and support the system until the malignant influence should have passed away; taking care to guard against hyperemia of internal organs, which constitutes the main danger of febrile diseases.

In the case now presented to the class,—the history of which follows,—certain of the phenomena of true typhoid, as delineated by Louis, Andral, and others, are as well marked, perhaps, as they ever are in any single case, and therefore adapted to impress the characters of the "typhoid affection" the more strongly upon the class.

Elizabeth M——, æt. 27, entered the wards January 21. Had been attacked by a chill, which was followed by fever, with pain in the head. Pulse 80, small and feeble; respiration nearly natural; tenderness and gurgling sound on pressure in right iliac fossa; slight resonance indicated by percussing the abdomen; slight meteorism; a mercurial fœtor was observed, without any mercury having been administered whilst in the hospital. Two days since, presented evidences of gastro-enteritis, which have gradually disappeared. At this time, the tongue appears slightly furred in the centre; skin moist, but not very hot; great tenderness, and gurgling upon pressure in the right iliac fossa; pulse 80, small and feeble; meteorism scarcely at all marked; *taches rouges* very distinct upon the abdomen, so as to be readily seen by the class.

Was first treated by an emeto-cathartic, and afterwards by mild cathartics; the indication being to keep the intestinal canal clear, but by no means to irritate it by violent purging. For this purpose, the Professor has been in the habit of using the Oleum Ricini, in teaspoonful doses, which he has generally found to be sufficient, and which he can recommend, not only in this, but in all other cases in which there is a morbid condition of the lining membrane of the bowels, and a similar indication has to be answered. Sponging the surface with tepid or cold vinegar and water, (the latter being equally effectual when used alone,) will be found to exert a favourable refrigerant influence, whenever the skin is steadily hot and dry, far exceeding any of the reputed diaphoretic agent so much in use. The Professor would trust little, in these cases, to the shop of the apothecary. He would keep the patient free, as far as possible, from all disturbing influences, as light, noise, &c. It may become necessary, in protracted cases, to excite a new action in the system by touching the mouth with mercury, or to give, as it were, a gentle fillip to the intestinal surface by means of some agent, as the Oleum Terebinth., which may be combined with the Oleum Ricini with good effect. External treatment may also be advisable, as cups, or other counter-irritants, to the abdomen; supporting the system in the latter periods, when necessary, by mild excitants, as wine whey, &c. &c. Under this simple treatment, more decided benefit will often be experienced than from any other course. Such has been the result of the Professor's experience, not only in this, but in the bilious remittent fevers of the country. Such, too, appears to be the result in the case at present under consideration, which bids fair, in the absence of any serious complication, to eventuate as favourably as could be desired.

### TUBERCULOSIS OF THE LUNG.

One other case the Professor desired to present to the class, for the purpose of more strongly impressing them with a due sense of the value of physical signs, and urging upon them the utility of making them the objects of particular study and attention. The case before the class exhibited the most marked difference between the two sides of the chest. It was therefore excellent for the young beginner, and the Professor said he would be glad if any of the class would step over, and examine the case for themselves, of which opportunity many gladly availed themselves.

The history of this case was as follows, Paul C. æt. 35, born in Ireland,—miller,—had been intemperate some years ago,—has since had an attack of dysentery, from which, however, he recovered,—was seized in May last with cough and deafness, for the former of which he was treated in the Baltimore Almshouse. When he entered the wards he complained of cough and acidity of the stomach; the latter has been relieved; expectoration from cough not great, but slightly increased in the mornings. Patient presents a cachectic appearance. His hair and eyes are dark, like those of many of the cachectic (tuberculous) cases which have come under the Professor's notice, as remarked upon a late occasion. On inspection, the chest was observed to rise more upon the left than the right side during inspiration. On percussion, great dulness over the whole upper part of the right side of the chest was observable. Auscultation indicated tubal or bronchial respiration with marked bronchophony on the same side. The inferences to be drawn from the above examination are—*first*, from the left side rising most during inspiration, that the lung of that side is performing the greater part of the function. *Secondly*, from the greater dulness of the right side as compared with the left, that consolidation has taken place there. It should always be borne in mind, in these examinations, that the difference in the two sides of the chest is that upon which diagnosis is to be chiefly founded; for although there may be tuberculous deposits in both lungs at the same time, this is by no means a frequent occurrence. *Thirdly*, from the tubal respiration in the right lung, it may be inferred, that the inspired air does not pass into the air cells, but remains in the bronchial tubes until expelled by expiration; and *lastly* the bronchophony or increased resonance of the voice would denote the existence of some better conductor of the vibrations of sound from the larynx to the ear of the listener, than is afforded by the healthy lung. From the laws which govern the vibrations of sound, we are aware, that the greater the solidity of a body, the better is it adapted for the transmission of sonorous vibrations, and from tihs again the solidification of the lung may be argued. The lecturer stated, however, that in the comparison of the sounds of the two sides of the chest a difficulty had presented itself to the mind of a German auscultator, Skoda, based upon the observed consociation of sounds. If we sound the note A upon a flute, the corresponding string A of a neighboring piano will be thrown into vibration by this reciprocation or consociation of sound. In like manner Skoda supposes there may be a condition of lung which may admit of being more readily thrown into reciprocal vibration without our inferring that condition to be one of consolidation. The Professor referred to cases observed by Skoda and also by himself, in which there was unusual bronchophony without there being reason to believe that consolidation existed. In this case, however, there is doubtless consolidation, inasmuch as there is not only bronchophony, but dulness on percussion, and abence of vesicular respiration. The consolidation, he said, would, probably, in this case, be succeeded by softening, some evidences of which are already present, and it would terminate as such cases usually do, in death.

### CONCLUSION.

Having terminated his observations on this case, the Professor proceeded, from a list which he had prepared, to make a brief recapitulation of the various diseases and their treatment, which had been considered during the clinical session. From this review it appeared that the course had embraced almost all the prominent affections, or those of every day occurrence in private praçtice, and more particularly those of a chronic character, which he thought the most difficult of treatment, often baffling the skill of the most experienced, generally requiring all the resources at his command, and always extremely trying to the young practitioner.

Under the head of DISEASES OF THE ALIMENTARY CANAL, have been presented,

| | |
|---|---|
| Chronic Gastritis, | Perforation of Intestine, |
| Hæmatemesis, | Tympanitis, |
| Endo-enteritis, | Chronic Peritonitis. |

DISEASES OF RESPIRATORY ORGANS.

| | |
|---|---|
| Laryngitis, | Pneumonitic abscess, |
| Bronchitis, | Tuberculosis in every possible stage, |
| Hæmoptysis, | |
| Pneumonia, acute, | Emphysema of the lungs, |
| ——— typhoid form, | Asthma, |
| Pleurisy, acute and chronic. | |

DISEASES OF CIRCULATORY APPARATUS.

| | |
|---|---|
| Anæmia, | Aortitis, |
| Hypertrophy of Heart, | Aneurisms of Aorta, |
| Softening of Heart, | ——— of Internal Iliac. |
| Diseases of Valves, | |

DISEASES OF GLANDIFORM GANGLIONS.

| | |
|---|---|
| Hypertrophy of Spleen, | Mesenteric Ganglionitis. |

DISEASES OF GLANDULAR ORGANS.

| | |
|---|---|
| Ptyalism, mercurial, | Ptyalism from Paralysis. |

DISEASES OF NERVOUS APPARATUS.

| | |
|---|---|
| Encephalitis, | Encephalic Hemorrhage, |
| Meningitis, | Ramollissement of the Brain, |
| Injury of Sensory Tract of Spinal Marrow, | Acrodynia, |
| Injury of Motor Tract of Spinal Marrow, | Epilepsy, |
| | Chorea, |
| Neuralgia. | |

FEVERS.

| | |
|---|---|
| Intermittent, | Remittent, |
| Typhoid. | |

ARTHRITIC FEVERS.

Rheumatism.

ERUPTIVE FEVERS.

Erysipelas.

(The contagious exanthemata, the Professor has refrained from presenting from obvious reasons.)

CACHEXIÆ.

| | |
|---|---|
| Hydropic, | Scrofulous. |

Thus, it will be seen that the cases presented comprise the greater part of the diseases ordinarily met with, and many of which were embarrassing as regards their correct diagnosis and treatment. The Professor drew the attention of the class to the fact, that although he had been desirous of exhibiting to them examples of bilious and renal diseases, yet that no case of the kind had presented itself in the wards during the session, a circumstance, which would lead them to hesitate before they adopted the views of those who were looking to the liver as the source of most maladies. He believed, that this rarity of cases of hepatic mischief was greatly owing to the temperance reformation.

In conclusion, the Professor made some parting remarks, and stated, that if the clinical instruction, which he and his colleague had endeavoured to communicate, should in future tend in any degree to smooth the paths of the young practitioners, they would consider themselves amply repaid.

—

As one of the great number who have availed themselves of the benefit derivable from clinical instruction at this Institution, (the Philadelphia Hospital,) it is but an act of justice, at the close of the session to state, that amidst the numerous advantages possessed by this city, over every other in the Union, in the opportunities afforded for such instruction, none ranks higher than this. The care, attention and well known ability of Professor Dunglison in selecting cases, together with the fidelity and skill with which they are represented and treated, added to the untiring philanthropy exhibited by him, as well as by his colleague, Professor Pancoast, in their disinterested exertions for the benefit of their class, as well as of suffering humanity, merit for these gentlemen the admiration and warm gratitude of all.

EDWARD R. SQUIBB.

